# An Informatics Approach to Reading the Label: Identifying Common Chemical Mixtures in Personal Care Products

**DOI:** 10.1289/ehp.124-A149

**Published:** 2016-08-01

**Authors:** Carol Potera

**Affiliations:** Carol Potera, based in Montana, also writes for *Microbe*, *Genetic Engineering News*, and the *American Journal of Nursing*.

For many years chemical risk assessments focused on exposures to single agents, but researchers are now paying more attention to chemical mixtures. Of particular interest are mixtures that people encounter in daily life, including combinations of ingredients in shampoo, deodorant, toothpaste, and other personal care products. In this issue of *EHP*, researchers describe a new informatics approach to identify chemical mixtures commonly found in personal care products.^1^

Some ingredients used in personal care products are associated with adverse effects in people or animals. For instance, there is evidence that some fragrance compounds and antimicrobials can exacerbate asthma.^2^ ^,^ ^3^ Other ingredients have shown endocrine-disrupting activity in animal studies—for instance, inhibition of testosterone production,^4^ suppression of thyroid hormone,^5^ and estrogen mimicry^6^ ^,^ ^7^ ^,^ ^8^ ^,^ ^9^—although effects in humans are unclear. Over time, a typical morning hygiene routine can result in cumulative exposures to multiple ingredients that can potentially have adverse effects singly or in combination.^10^

**Figure:**
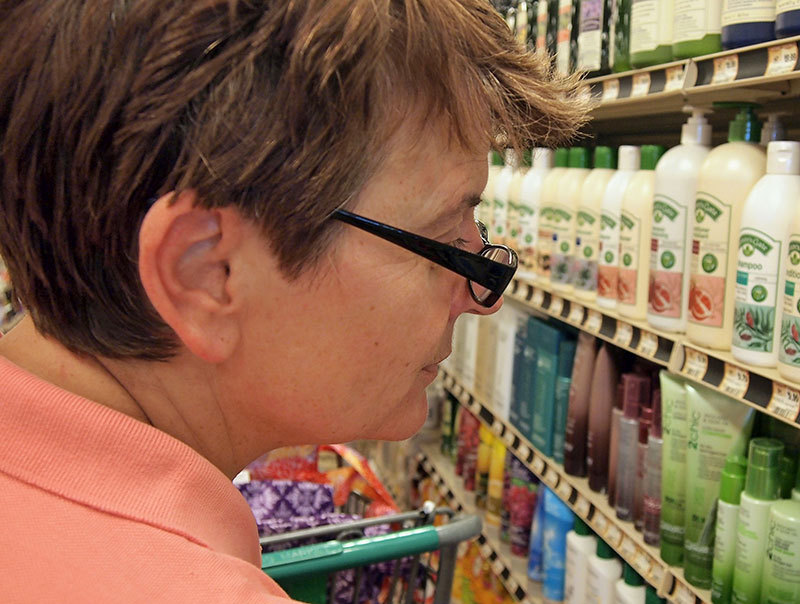
Consumers are sometimes limited in their ability to determine whether products contain chemicals they wish to avoid. © Katharine Andriotis/Alamy Stock Photo

People with allergies, asthma, and other conditions may rely on product labels to make informed decisions about the items they use. But many products only list “fragrance” or “flavor” on the ingredient label instead of specific chemicals comprising that fragrance or flavor.

Chemicals can also go by multiple names, making it difficult for consumers to interpret labels. For example, bucinal, a common synthetic fragrance ingredient, may also appear under its synonyms lilial or butylphenyl methylpropional. “Even a chemist would have a difficult time remembering all the different names for a chemical ingredient,” says study coauthor Henry Gabb, a research assistant in the University of Illinois at Urbana–Champaign School of Information Sciences.

The current study focused on 55 potentially problematic chemicals that an earlier study had quantified in personal care products.^11^ Gabb and coauthor Catherine Blake, an associate professor in the School of Information Sciences, used an informatics approach to develop a database of consumer products. This involved special software that they used to collect product ingredient information from online retailer Drugstore.com, creating a database of 38,975 distinct products. Then they parsed the ingredient information to identify chemicals that occurred frequently in the products, either singly or in mixtures. They used the Unified Medical Language System and the PubChem Compound database to match up chemical synonyms.

When the authors examined the product labels to see which ones contained any of the 55 target chemicals, they found that 30% contained at least 1 target chemical, while 13% contained more than 1. At least 1 of the target chemicals occurred in 70% of sunscreens, 69% of eye makeup products, 66% of lotions, 58% of conditioners, 44% of shampoos, 42% of lipsticks, 33% of body washes, 12% of deodorants, and 12% of toothpastes. More than a third of the target chemicals were listed by different names on different labels.^1^

The most commonly occurring chemicals were the preservatives 2-phenoxyethanol and methyl paraben, the fragrance compounds limonene and linalool, and the ultraviolet filter octinoxate. These chemicals often occurred as pairs or trios in consumer products, although the most frequently occurring trio—2-phenoxyethanol, methyl paraben, and ethyl paraben—was found in just 3% of the products.^1^

The authors point out that missing or incomplete product labels can limit how much data an informatics approach can retrieve. Still, the results indicate that publicly available data can be useful in identifying chemical mixtures that people are often exposed to. This information could help guide future toxicological and epidemiological research.

“Our paper underscores why it’s important to have ingredient lists that actually show what’s in a product, and in a language that consumers can understand,” Blake says. “I also hope our work prompts further discussions about what should or should not be on product labels.”

“This research is an important addition to the growing literature on consumer product chemicals. The study addresses some significant knowledge gaps related to consumer product chemical exposures,” says Robin Dodson, a research scientist at the Silent Spring Institute, who was not involved in the current study. “More complete product ingredient labeling, supplemented with actual product testing, will help consumers avoid certain chemicals.”
